# Glycerol Monolaurate Microbicide Protection against Repeat High-Dose SIV Vaginal Challenge

**DOI:** 10.1371/journal.pone.0129465

**Published:** 2015-06-09

**Authors:** Ashley T. Haase, Eva Rakasz, Nancy Schultz-Darken, Karla Nephew, Kimberly L. Weisgrau, Cavan S. Reilly, Qingsheng Li, Peter J. Southern, Meghan Rothenberger, Marnie L. Peterson, Patrick M. Schlievert

**Affiliations:** 1 Department of Microbiology and Immunobiology, Medical School, University of Minnesota, Minneapolis, MN 55455, United States of America; 2 Wisconsin National Primate Research Center, University of Wisconsin, Madison, WI 53715, United States of America; 3 Division of Biostatistics, School of Public Health, University of Minnesota, Minneapolis, MN 55455, United States of America; 4 Division of Infectious Diseases, Department of Medicine, Medical School, University of Minnesota, Minneapolis, MN 55455, United States of America; 5 Department of Experimental and Clinical Pharmacology, College of Pharmacy, University of Minnesota, Minneapolis, MN 55455, United States of America; University of Pittsburgh, UNITED STATES

## Abstract

Measures to prevent sexual mucosal transmission are critically needed, particularly to prevent transmission to young women at high risk in the microepidemics in South Africa that disproportionally contribute to the continued pandemic. To that end, microbicides containing anti-retroviral (ARV) agents have been shown to prevent transmission, but with efficacy limited both by adherence and pre-existing innate immune and inflammatory conditions in the female reproductive tract (FRT). Glycerol monolaurate (GML) has been proposed as a microbicide component to enhance efficacy by blocking these transmission-facilitating innate immune response to vaginal exposure. We show here in an especially rigorous test of protection in the SIV-rhesus macaque model of HIV-1 transmission to women, that GML used daily and before vaginal challenge protects against repeat high doses of SIV by criteria that include virological and immunological assays to detect occult infection. We also provide evidence for indirect mechanisms of action in GML-mediated protection. Developing a sustained formulation for GML delivery could contribute an independent, complementary protective component to an ARV-containing microbicide.

## Introduction

Combination antiretroviral therapies (ART) have had a highly beneficial impact on those already infected with HIV [1], but prevention of sexual mucosal transmission, particularly to the women who bear the brunt of infection in the pandemic’s epicenter in Africa [2], [3], is still urgently needed to stem the progress and ultimately end the pandemic. To that end, there have been encouraging results using ARV treatment as prevention in pre-exposure prophylaxis [4] and as a microbicide, where a 1% tenofovir (TFV) gel used before and after sex reduced acquisition of HIV on average by 39%, and by greater than 50% in women who used the gel more than 80% of the time [5].

It is now clear, however, from the subsequent disappointing failure in the VOICE trial to reproduce these protective effects of a TFV microbicide [6] that adherence plays a critical role in the success or failure of these ARV-based microbicides. If the microbicide is used inconsistently, drug levels in the female reproductive tract (FRT) will be too low for protection [7]. Moreover, in the post-trial analyses of the TFV microbicide, it is also clear that pre-existing innate immune and inflammatory conditions in the FRT play important roles in microbicide efficacy [8], in addition to the known role of genital tract inflammation in general in increased transmission [9], [10].

The relationship between pre-existing inflammation in the FRT, acquisition and microbicide efficacy points to the possibility that incorporating agents that modulate the innate and inflammatory *milieu* might enhance the efficacy of ARV microbicides, as well as prevent transmission by a mechanism independent of blocking viral replication. Glycerol monolaurate (GML) is an example of such a candidate to combine with ARVs in a microbicide that is attractive for two reasons: 1) GML is a fatty acid monoester that stabilizes membranes [11] and is thought in this way to block bacterial pore-forming toxins and T cell activation [12], [13]; and 2) GML blocks expression of pro-inflammatory cytokines and chemokines such as MIP3-alpha in epithelium in vitro and in vivo to thereby inhibit recruitment of CD4^+^ T cells to the FRT of rhesus macaques [14]. Thus GML could block transmission by inhibiting chemokines and cytokines that facilitate transmission through active recruitment of susceptible CD4^+^ target cells that fuel expansion of infected founder populations at the portal of entry.

In support of GML’s potential as a microbicide, we previously showed that GML was safe for long-term topical application to the FRT of rhesus macaques [15]; that GML reduced expression of transmission promoting cytokines and chemokines [14]; and potentially by this mechanism could protect against an extremely rigorous vaginal challenge with high doses of > 10^9^ copies of SIV RNA/ml. These high doses exceed the level of HIV-1 in semen in the range of 10^5^ to 10^7^ copies of HIV-1 RNA/mL where most transmissions occur [16], and this model is thus an especially rigorous test of protection. Under these stringent conditions to demonstrate efficacy, five of five GML-treated animals had undetectable plasma viral load (VL) 14 days after exposure to as many as four inoculations with 10^9^ copies of SIVmac251 RNA, whereas only one of five controls was protected against two inoculations and none of the five animals was protected against four challenges [14].

While this pilot study provided evidence of GML’s promise as an effective microbicide on its own, it was limited in the relatively few animals studied. Moreover, one GML-treated and apparently protected animal at peak replication at 14 days post vaginal challenge, nonetheless surprisingly had evidence of systemic infection at five months after the last vaginal challenge [14]. We therefore undertook and report here a larger study of GML efficacy, with long term and more extensive evaluation of occult infections by assaying SIV-specific T cell responses [17], [18].

## Materials and Methods

### Animals

We followed the National Research Council Guide for the Care and Use of Laboratory Animals for the work utilizing the 26 female rhesus macaques and received approval from the University of Wisconsin Graduate School IACUC with permit G00513. Monkeys were singly housed in standard stainless steel primate cages (Suburban Surgical, Chicago, IL). All females were of reproductive age and none were on progestin. The stage of the menstrual cycle did not differ between the GML and control groups, and was split evenly between the between follicular and luteal phases. All animals had visual and auditory contact with each other in the same room. They were fed twice daily with commercial chow (Harlan Teklad #2050, 20% protein Primate Diet, Madison, WI) and given a variety of fruit in the afternoons. In addition we provided foraging activities and physical environmental enrichment at least weekly for both activities. Housing rooms were maintained at 65–75°F, 30–70% humidity, and on a 12:12 light-dark cycle (ON: 0600, OFF: 1800).

### GML

A GML gel for vaginal use (GML Vaginal Gel) was prepared at the Fairview Minnesota Compounding Pharmacy, Minneapolis, MN [15]. Briefly, 5% (50 mg/ml) GML (Colonial Chemical, Inc, South Pittsburg, TN) was mixed with a non-aqueous gel by addition of the following pharmacy-grade excipients: propylene glycol (73.55%), polyethylene glycol 400 (25%), hydroxypropyl cellulose (1.25%), and lactic acid (if needed to adjust pH to equal 4.5). A Placebo Control Gel contained excipients without added GML. GML or placebo was administered daily with gentle intravaginal insertion of a 1ml syringe in awake, briefly restrained females. Immediately prior to the treatment, the surface of the vagina and anus were wiped with dilute chlorhexidine gluconate (1:1 water) to remove any surface debris. GML was also administered 1 hour prior to any SIV vaginal challenge.

### Collection of Cervical Vaginal Fluid (CVF) and Determination of GML and Cytokine Levels

CVF was collected using a weighed cotton swab inserted atraumatically into the vaginal canal for several minutes to collect cervicovaginal fluids. The swabs consistently and reproducibly absorbed 0.1 ml of CVF. For the pharmacokinetic (PK) studies of GML levels, 1 swab was collected just prior to and immediately after administering GML, and at approximately 4 and 8 hours after insertion, GML was extracted from samples with methylene chloride, and the GML-containing samples and controls, and standards with known amounts of GML dissolved in 60/40 methylene chloride, were assayed by GC/mass spectrometry for both GML and its breakdown product, lauric acid. For the PK studies of cytokines, swabs were obtained at baseline and 1 through 16 days after GML treatment. MIP3-alpha and IL-8 levels were measured by ELISA according to the manufacturer’s directions (R&D Systems, Minneapolis, MN).

### High Dose SIV Vaginal Challenge

Animals were challenged intra-vaginally with a SIVmac251 virus stock containing 10^5^ TCID_50_ and 10^9^ copies of viral RNA per ml [19]. Each high dose vaginal challenge consisted of two intra-vaginal inoculations of SIVmac251 in 1ml RPMI administered 4 hours apart. Animals were anesthetized with ketamine/dexmedatomidine (atimpamizole reversal) prior to each inoculation. The 1ml syringe was gently introduced into the vagina about 4 cm and withdrawn slightly before slow injection for 1 minute. After injection, the animal was maintained in a position to allow the inoculum to drain toward the cervix for approximately 40 minutes.

### Plasma Viral Load

Viral RNA was extracted from EDTA-anticoagulated plasma using guanidine hydrochloride as previously described [20], [21]. Viral RNA was extracted from some samples using the Qiamp UltraSens virus kit (Qiagen, Valencia, CA) or the PureLink Viral RNA/DNA mini kit (Life Technologies, Grand Island, NY). Viral RNA was quantified from plasma in a taqman assay using forward primer (SIV1552), 5’-GTCTGCGTCATCTGGTGCATTC-3’, reverse primer (SIV1635), 5’-CACTAGCTGTCTCTGCACTATGTGTTTTG-3’, and probe, 5’-6-carboxyfluorescein-CTTCCTCAGTGTGTTTCACTTTCTCTTCTGCG-6-carboxytetramethylrhodamine-3’. Reactions were performed with the SuperScript III Platinum One-Step Quantitative RT-PCR Kit (Invitrogen, Grand Island, NY) on the LightCycler 480 (Roche, Indianapolis, IN) as previously described [20].

### Flow Cytometric Analysis of SIV Antigen-Specific Cellular Responses

Intracellular cytokine staining was performed as previously described [22]. Briefly, 10^6^ freshly isolated PBMC were stimulated with 1μM pool of overlapping 15-mer oligopeptides covering either the first or second half of Gag (aa1-291, or 281–510), or the entire length of Nef (aa1-264) of SIVmavc251 at 37°C in 5% CO_2_ overnight. The stimulation mixture also included anti-CD28 (clone L293), anti-CD49d (clone 9F10) and anti-CD107a (PE conjugated, clone H4A3) antibodies. To prevent the secretion of cytokines, Brefeldin A (Sigma-Aldrich, St. Louis, MO), and GolgiStop (BD Biosciences, San Jose, CA) were added according to the manufacturer’s recommendation. Matching SEB stimulated samples provided positive, and no peptide stimulated samples provided negative control values. Following the stimulation cells were stained for the surface expression of CD8 (Pacific Blue conjugated antibody, clone SK1), and CD4 (PerCPCy5.5 conjugated antibody, clone SK3) antigens, washed twice with RPMI containing 10% fetal calf serum (R10), and fixed with 2% paraformaldehyde for at least 30 minutes at room temperature. Cells were then permeabilized with Saponin buffer (PBS containing 0.1% Saponin and 10% FCS), intracellularly stained for IFN-γ (FITC conjugated antibody, clone 4S.B3), and IL-2 (APC conjugated antibody, clone MQ1-17H12), washed twice with Saponin buffer, and fixed with 2% paraformaldehyde for 30 minutes at 4°C. All antibodies were obtained from BD Biosciences, San Jose, CA. 2–4 x 10^5^ events were collected within the lymphocyte gate using FACSDiva 6.2 software on a SORP BD LSR II flow cytometer (BD Biosciences, San Jose, CA) equipped with a 50 mW 405 nm violet, a 100 mW 488 nm blue, and a 50 mW 640 nm red laser. Data were analyzed using FlowJo 9.1 (TreeStar, Inc., Ashland, OR). Responses at least two times higher than the negative control values were considered positive.

### Statistical Analysis

Since the experimental protocol called for sequential re-challenge until all control animals were infected, one cannot estimate the efficacy and test for differences between groups using sample proportions. As in ref. [14], we instead use the negative binomial distribution to model the number of times required to infect an animal and suppose that the treated and control animals have group specific probabilities of infection. We then specify prior distributions for these probabilities that are uniform over the interval [0, 1] and find that the posterior distributions for the group specific probabilities are beta distributions with parameters 9 and 16 in the group treated with GML and 14 and 4 in the control group. By simulating draws from these distributions we can use Monte Carlo integration to estimate the posterior probability that the probability of infection is lower in the GML-treated group (see results).

## Results

### Experimental Design

Our principal goal in these studies was to assess protection conferred by GML against high-dose vaginal challenge in a substantially larger number of animals than previously reported [14]; and to follow animals for extended periods with virological and immunological analyses to determine whether animals ostensibly protected against acute infection were or were not harboring virus in an occult infection (OI). Overall, 13 rhesus macaques were treated (Rx) with 5% GML in a non-aqueous gel resembling K-Y Warming Gel, referred to as GML Vaginal Gel, versus 13 animals in the vehicle control cohort, treated with only the Placebo Control Gel. Based on the protocols used in the pilot study [14] and the results of studies described below, 1 ml doses of GML were administered vaginally every day for at least two weeks before the first challenge, and, on the day of challenge, animals were treated with GML or the vehicle control 1 hour prior to atraumatic vaginal inoculation of 10^9^ copies of SIVmac251 RNA; and again 1 hour before the same dose of virus on the same day four hours later, as previously described in this high-dose vaginal challenge model [19]. Daily treatments were then continued. VLs were monitored for evidence of systemic infection (VL of 50 copies/ml, the limit of detection-LOD) for at least a month, based on previous studies in which acute infection with high VLs had been manifest by 28 days [19]. Animals that did not have detectable VL at 28 days that met this criterion for protection were re-challenged. To assess occult infection in animals that met the protection criterion in GML-cohorts 1 and 2 (Table 1), treatment and VL monitoring was continued for >365 days. Animals in cohort 3 were continued on treatment and followed for extensive periods after the first challenge for immunological assays of OI.

**Table 1:** Administration of GML (or placebo) for all study animals was once per day, except on each challenge day when treatment was given 1 hour prior to each of two separate inoculations of SIVmac251, resulting in two doses.

**GML**	**Days preRx**	**Days VL nd and Rx post-Challenge 1**	**Days Rx post-Challenge 2**	**Days VL nd post-Challenge 2**	**Infected**
r99018	14	36	119	>365	**N**
r99044	14	36	119	>365	**N**
r99062	14	36	267	>365	**N**
r03056	21	34	262	>365	**N**
r04071	21	34	262	>365	**N**
r06017	21	34	262	>365	**N**
r05064	21	34	214	n/a	Y
r02037	16	266	23	n/a	Y
r03147	16	0	n/a	n/a	Y
r04073	16	266	181	>365	**N**
r04119	16	0	n/a	n/a	Y
r04129	16	0	n/a	n/a	Y
r04133	16	266	23	>365	**N**
**Placebo only**	**Days preRx**	**Days VL nd and Rx post-Challenge 1**	**Days Rx post-Challenge 2**	**Days VL nd post-Challenge 2**	**Infected**
r01084	14	0	n/a	n/a	Y
r98058	14	36	82	0	Y
r98060	14	36	119	0	Y
rh2148	16	0	n/a	n/a	Y
r04061	21	34	34	0	Y
r05044	21	0	n/a	n/a	Y
r05048	27	0	n/a	n/a	Y
r07001	27	0	n/a	n/a	Y
r02100	16	0	n/a	n/a	Y
r02125	16	0	n/a	n/a	Y
r03038	16	0	n/a	n/a	Y
r03066	16	0	n/a	n/a	Y
r03070	16	0	n/a	n/a	Y

There were 3 cohorts consisting of both treatment and control animals indicated by degree of shading (cohort 1 = lightest; cohort 2 = medium; cohort 3 = darker). Cohorts only differed in the scheduled time of pre-treatment and first and second challenge days. All cohorts received the same dose continuously for the time indicated. In addition, viral loads for uninfected animals were followed for at least one year after challenge 2.

### GML Protection Against Repeated High-Dose Vaginal Challenge

**Fig 1:**
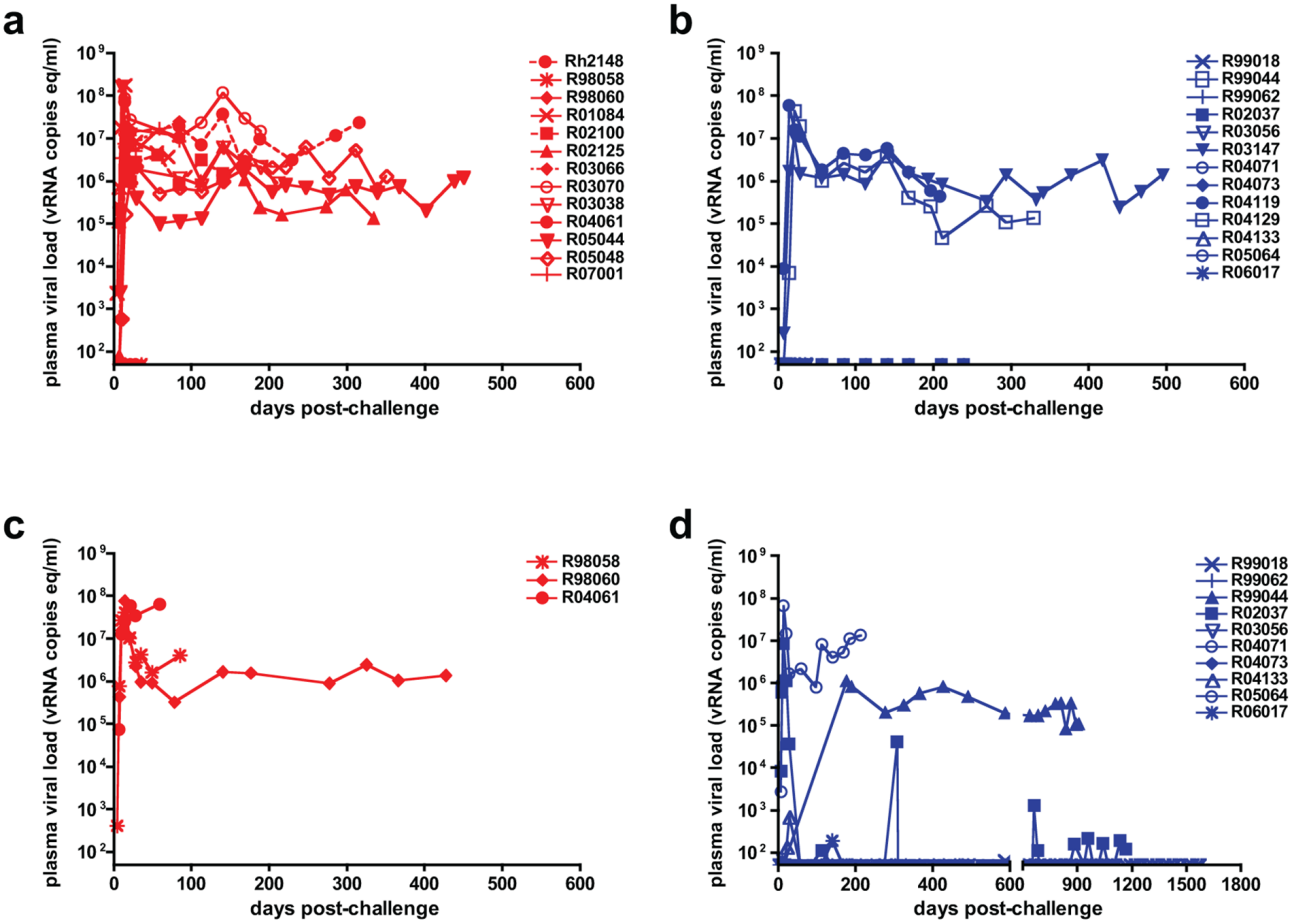
GML Protection Against High-Dose Vaginal Challenge. *A*, 13 vehicle-control animals (identifying numbers shown in each panel) and *B*, 13 GML-treated animals were challenged vaginally with 2 doses the same day of 10^9^ copies of SIVmac251 RNA. *C*, *D*, Uninfected animals were re-challenged and followed for more than one year if viral loads were undetectable. Plots stop when animals were euthanized for clinical symptoms.

The high-dose vaginal challenge model of transmission of two separate inoculations the same day of 10^9^ copies of SIVmac251 RNA has been reported to infect 15 of 16 rhesus macaques [23]. In reasonable agreement with that report, high-dose vaginal challenge infected 10 of 13 animals in the vehicle control cohort and the remaining 3 animals on re-challenge (Fig 1A,1C). VL peak and set points were typical for robust systemic infections, and thus the control group achieved the pre-defined endpoint of the study of robust systemic infection in all 13-control animals with two challenges. By contrast, the first high-dose vaginal challenge resulted in systemic infection of only 3 of 13 animals in the GML-treated cohort and infection on re-challenge of 4 of 10 animals. Moreover, 2/4 of the GML-treated animals were non-progressors with low-level viremia or detectable VL on only one measurement (r04133) (Fig 1B,1D). Statistical analyses of these results estimate a mean of protection of 0.640 in the GML-treated group and 0.222 in the control group. Moreover, the probability that the chances of infection are lower in the GML-treated group is 0.9978, providing near certainty that GML treatment lowers the chances of infection in this model. We also find minimal width 95% credible sets for the probability of protection in each group (these are similar to 95% confidence intervals). These intervals are (0.455, 0.819) in the GML-treated group and (0.053, 0.410) in the control group.

**Fig 2:**
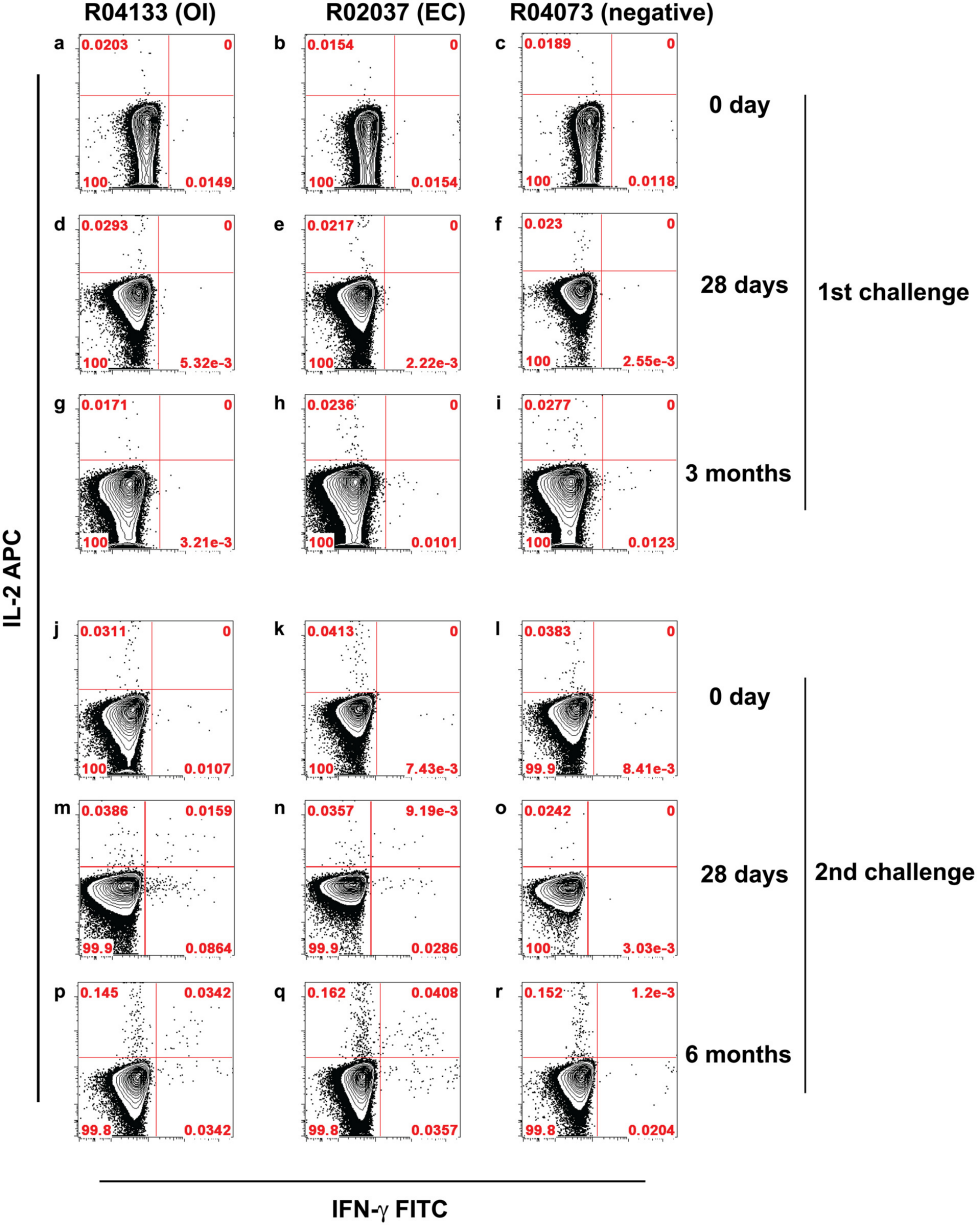
CD4 T Cell Responses Against SIVmac251 Gag After the Second Viral Challenge. Freshly isolated PBMC were stimulated with overlapping 15-mer oligopeptides of SIVmac251 Gag aa1-291 and stained for the presence of intracellular cytokines IFN-gamma and IL-2. Bivariate plots display events gated for CD4 antigen expression within the lymphocyte gate. Comparison from simultaneously acquired data from animals classified from their VL as OI (R04133: VLs of 1.37 and 7.08 x 10^2^ copy eq/ml at respectively weeks 3 and 4 post second challenge; VLs thereafter below LOD), elite controller animal (R02037: peak viral load at week 2 post-infection is 8.46 x 10^6^ vRNA copy eq/ml, followed by set-point viral load below detection level from eight week post-infection), and an uninfected animal (R04073). Frequency of IFN-gamma and/or IL-2 positive CD4^+^ lymphocytes at *a-c*, day 0; *d-f*, day 28; and *g-I*, 3 months after the first viral challenge. Frequency of IFN-gamma and/or IL-2 positive CD4^+^ lymphocytes at *j-l*, day 0; *m-o*, day 28; and *p-r*, 3 months after the second viral challenge.

**Fig 3:**
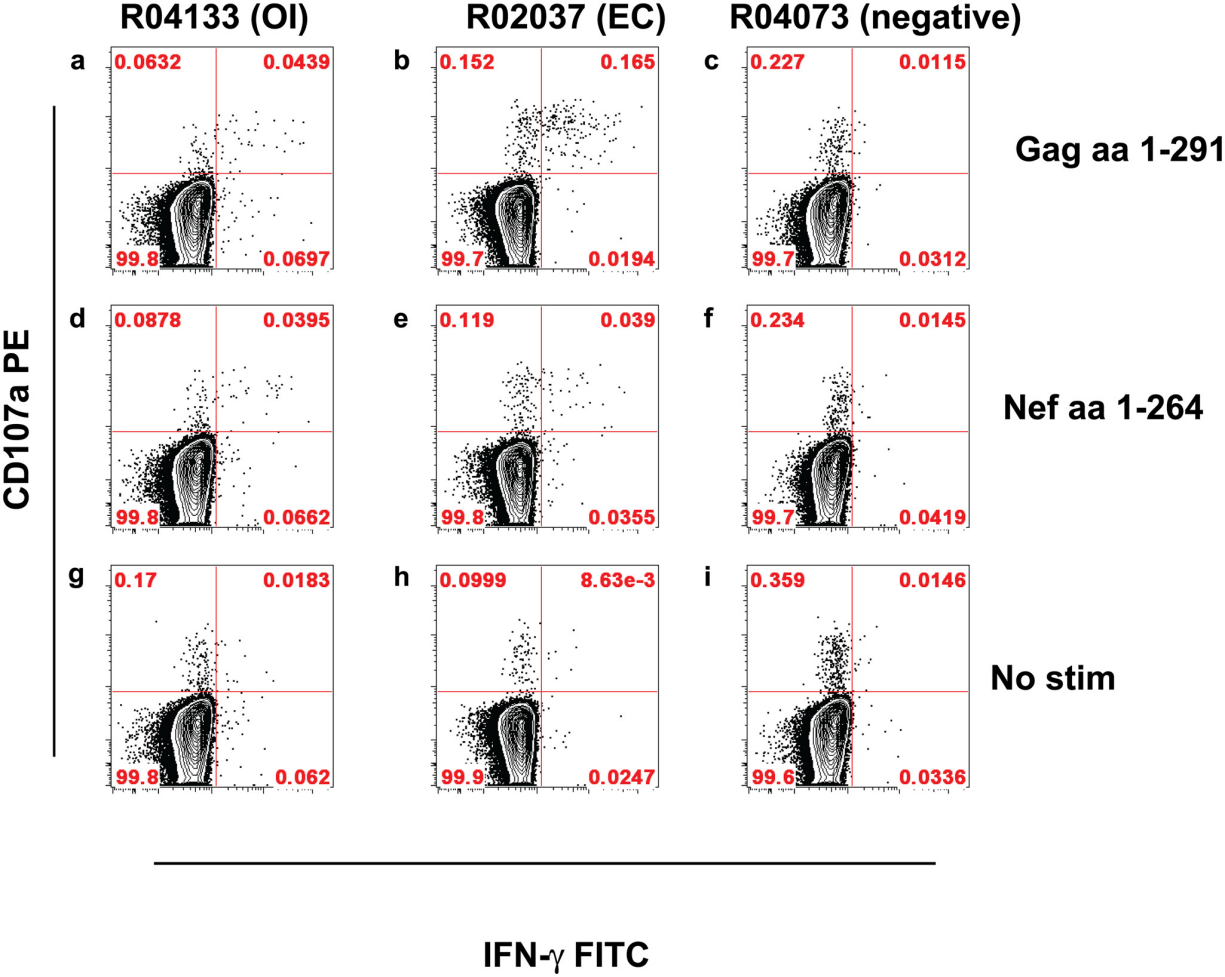
The SIVmac251-Specific CD8 T Cells of Animal R04133 (OI) at 6 Months Following the Second Challenge Possess Cytotoxic Capability. Freshly isolated PBMC were stimulated with either overlapping 15-mer oligopeptides of SIVmac251 Gag aa1-291 (*a-c*) or Nef aa1-264 (*d-f*) and stained for IFN-gamma and CD107a. Plots *g-I*, show simultaneously measured background data. Bivariate plots display events gated for CD8 antigens within the lymphocyte gate.

The 6 GML-treated/protected animals on re-challenge were monitored for more than one year for evidence of OI (Cohorts 1 and 2, Table 1). None of the protected animals had VLs above the LOD. We also sought to detect OI by monitoring SIVmac251 Gag and Nef-specific cellular responses in a subset of the animals after the first challenge; and, after the second challenge, the immune response in the EC animal and animal with OI (Cohort 3). None of the animals with persistently undetectable viral load had measurable CD8 T cell responses up to six months following the first viral challenge (Table 2). For positive controls for the intracellular cytokine staining (ICS) assays, we used animals that were viremic and, as expected, these animals developed strong T cell responses by one month post-challenge. In the animal with detectable VL on one measurement (r04133), we documented small but reproducible T cell responses against both Gag and Nef; and in the other animal with very low VLs (r02037) we detected CD4^+^ subsets secreting IL-2 and IFN-gamma (Fig 2); and CD8^+^ IFN-gamma^+^ subsets with cytolytic capability, as defined by the presence of CD107a antigen expression (Fig 3).

**Table 2:** SIVmac251-specific CD8 T cell responses in selected animals of the study.

Challenge	Group	Animal ID	Viral status	Gag A-G			Nef		
				Months post-challenge					
				1	3	5–7	1	3	5–7
First	Control	R05044	Positive	+	ND^a^	-	+	ND	+
First	Control	R05048	Positive	+	ND	+	+	ND	+
First	Control	R07001	Positive	+	ND	ND	+	ND	ND
First	GML-treated	R02037	Negative	-	-	-	-	-	-
First	GML-treated	R03056	Negative	-	-	-	-	-	-
First	GML-treated	R04071	Negative	-	-	-	-	-	-
First	GML-treated	R04073	Negative	-	-	-	-	-	-
First	GML-treated	R04133	Negative	-	-	-	-	-	-
Second	GML-treated	R02037	EC^b^	+	+	+	+	+	+
Second	GML-treated	R03056	Negative	-	-	-	-	-	-
Second	GML-treated	R04073	Negative	-	-	-	-	-	-
Second	GML-treated	R04133	OI^c^	+	+	+	+	+	+

^a^ND: Not Done

^b^EC: Elite Controller

^c^OI: Occult Infection.

### GML Pharmacokinetics and Indirect Mechanism of Action

**Fig 4:**
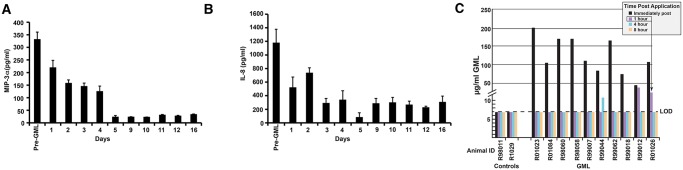
GML Pharmacokinetics (PK) and Indirect Mechanism of Action. *A*, *B* PK of daily administration of GML on suppressing MIP-3 alpha and IL-8 levels in CVF. Means and SD indicated for three animals. *C*, GML levels in CVF in treated animals and controls immediately after application and 1, 4 and 8 hours later. Arrow indicates the level prior to challenge at the one-hour time point in animal R01026. LOD = Limits of Detection = 7ug/ml, indicated by a dotted line. The Y-axis scale is discontinuous to show levels of GML immediately after administration.

The pilot protection study showing efficacy against high dose vaginal challenge [14] was initiated in animals immediately after they had been treated daily with GML for months to document the safety of chronic topical exposure [15]. We now asked whether this fortuitous sequencing of the extended pre-treatment with GML before challenge might have been critical for protection by blocking MIP-3 alpha and IL-8 production [11]-[14] to thereby reduce availability of the CD4^+^ target cells that fuel local expansion of infected founder populations at the portal of entry [24]. Given the primary objectives of the study to show protection against vaginal challenge, we could not collect tissue samples to directly test this hypothesis, but we did determine in three animals the PK in the CVF of suppression. GML maximally reduced levels of MIP-3 alpha and IL-8 in CVF by 13 to 15 fold compared to pre-GML levels (Fig 4A and 1B), but these reductions required 5 days of pre-treatment, consistent with the idea that pre-treatment was critical for protection. Based on these results, we designed the efficacy trial as described above with a pre-treatment phase of two weeks or more.

The necessity of pre-treatment also suggested that GML’s mechanism of action was indirectly mediated by suppressing the cytokines that facilitate infection in the female reproductive tract (FRT) rather than direct inactivation of virus. We therefore measured the levels of GML in CVF immediately after administration and 1, 4 and 8 hours later and then compared those levels with those reported to directly inactivate HIV-1. Even immediately after administration, the levels in CVF (Fig 4C) were at least 5-fold lower than the 0.1% reported to directly inhibit HIV-1 replication in vitro [25]. By 1 hour, the time of vaginal exposure to SIV, presumably because of the observed leakage of gel, the levels were 50 to more than 100-fold lower than the concentration needed to directly affect virus in the inoculum.

## Discussion

This larger and more extensive study of GML as a topical microbicide documents its previously reported [14] efficacy against repeated high-dose vaginal challenge in the SIV-rhesus macaque non-human primate model of HIV-1 transmission to women by two criteria: 1) VLs consistently below the LOD for more than a year; and 2) no evidence immunologically of OI [17], [18] in protected animals. We chose this exceptionally rigorous high dose challenge, rather than repeat low dose exposures, for two reasons. First, there is a strong case that prevention strategies need to target the microepidemics in South Africa and elsewhere that continue to drive the pandemic [26] as a consequence in part of exposure to the high levels of HIV [16]. Second, transmission in these “hot zones” is often associated with co-existing innate immune activation in the FRT [8] that the high dose model mimics [14] and GML targets [14]. The 2x10^9^ copies of SIV RNA in our challenges was two orders of magnitude higher than the highest semen levels of 10^7^ copies of viral RNA/ml associated with high rates of transmission, and should infect all of the controls with a limited number of challenges, an important logistical consideration in the design of nonhuman primate studies. For comparison, while topical integrase inhibitors have recently been reported to protect 5/6 macaques against repeat low dose vaginal challenges [27], the challenge doses used were ~ 1000 fold lower than the ones we used in the high dose challenges. From the perspective of targeting prevention to high-risk populations, GML was thus effective in preventing transmission against four exposures to 10^9^ copies of SIV RNA in about half of the animals.

While the immunological analyses were primarily focused on detecting OI, the immunological response in the two re-challenged GML-treated animals that controlled infection is intriguing. In both animals, we document multifunctional responses in both CD4^+^ and CD8^+^ T cells that correlated with low VLs. The results are consistent with the speculation that GML suppression of the innate immune response to vaginal exposure to SIV might have sufficiently disrupted local expansion to delay and reduce systemic infection. This would then provide an opportunity for the host to mount an immunological response that is better able to control systemic infection.

The several days needed to suppress the chemokines that mediate target cell recruitment and a milieu of innate immune activation are consistent with the hypothesized indirect mechanism of action in preventing transmission. The levels of GML just prior to challenge were many-fold lower than the levels necessary to directly inactivate HIV-1 in vitro and therefore also consistent with an indirect mechanism of action. Thus GML could potentially complement an ARV-containing microbicide to provide an independent and cooperative mechanism of inhibiting viral replication at the portal of entry. The current clear limitations to this strategy are the requirement for daily dosing and the relatively high concentration of GML and volume associated with leakage. Sustained release formulations would need to be developed to deliver high concentrations of GML with minimal loss for GML to be an effective microbicide against vaginal transmission of HIV-1.
